# Announcing *Signal Transduction and Targeted Therapy*

**DOI:** 10.1038/sigtrans.2015.6

**Published:** 2016-01-28

**Authors:** C M Croce, K Zhang, Y-q Wei

**Affiliations:** 1 Institute of Genetics, The Ohio State University, Columbus, OH, USA; 2 Shiley Eye Institute, Institute for Engineering in Medicine, Institute for Genomic Medicine, University of California San Diego, San Diego, CA, USA; 3 State Key Laboratory of Biotherapy, West China Hospital, Sichuan University/Collaborative Innovation Center of Biotherapy, Sichuan, China

Since the concept of signal transduction was introduced in the 1970s, various signal transduction pathways and signaling molecules have been identified. A major driving force in this field is the potential for signaling pathway-targeted therapy in clinical applications. Indeed, the fundamental research in cell signal transduction has led to the success of targeted therapies, such as imatinib (Gleevec; Novartis, Basel, Switzerland), for the treatment of chronic myelogenous leukemia, and trastuzumab (Herceptin; Roche/Genentech, South San Francisco, CA, USA), for the treatment of HER2 (human epidermal growth factor receptor 2)-positive breast cancer, which, in turn, has further stimulated basic research in signal transduction. Despite advances in our understanding of signal transduction and signaling pathway-targeted therapies, there are still many challenges to be faced. The cell signaling network and its regulation are more complicated than previously anticipated. Signal transduction between cells and between tissues is still far from understood. Constantly emerging gene mutations and drug resistance to targeted therapies indicate unmet clinical needs. Furthermore, the growing publication record indicates the field is rapidly expanding. Based on a PubMed Advanced Keyword Search—‘signal transduction and/or targeted therapy’—the total number of papers published in 1990 was just 3674, which has since risen to 15 225 in 2000, and 47 730 in 2013. However, high-impact professional journals related to this field are lacking, and, as a result, the published literature is vastly decentralized. Furthermore, governments around the world are increasing funding for research in *Signal Transduction and Targeted Therapy*, including basic science research, targeted drug research and development, and the clinical application of targeted drugs. Undoubtedly, there will be new and exciting discoveries in this field in the near future, and the limited capacity of existing journals cannot ensure the timely publication of relevant works that will emerge. In this light, we are establishing *Signal Transduction and Targeted Therapy* to consolidate and advance the growing body of knowledge in this field.

The scope of *Signal Transduction and Targeted Therapy* encompasses signal transduction in physiological and pathological processes, as well as signaling pathway-targeted therapies, including biological agents and small molecular drugs used in the management of human diseases. Areas of interest also include basic and translational research in molecular pharmacology and chemotherapy, drug sensitivity and resistance, tumor immunology and immunotherapy, biomarkers and prognostic indicators, gene therapy, cell adhesion, invasion and metastasis, differentiation and cell death, clinical genetics, medicinal chemistry, systems biology, pharmacy, nanometer materials, computational chemistry, and computational biology. The disease types span all the major human diseases, including cancer, immune disorders, diabetes, cardiovascular diseases, inflammation, central nervous system diseases and other pathologies.

A notable feature of *Signal Transduction and Targeted Therapy* is that it is a multidisciplinary journal, covering topics including but not limited to molecular biology, cell biology, pathology, medicinal chemistry, computational chemistry, systems biology, bioinformatics, pharmacology, pharmaceutics and clinical medicine. It has a wide range of authors and readership, including researchers engaged in basic research and applied research related to signal transduction and signaling pathway-targeted therapy, as well as clinicians.

We aim to be the leading forum for research on cell signal regulation, molecular abnormalities that predict incidence, response to therapy, and outcome; targeted drug discovery and preclinical studies of new drugs; and clinical trials evaluating new treatments for various diseases. In addition to original research articles and review articles related to *Signal Transduction and Targeted Therapy*, invited viewpoint articles may be aperiodically published, which address timely and controversial topics in cell signal regulation and targeted therapy.

Researchers are welcome to contribute valuable work to *Signal Transduction and Targeted Therapy*. We promise fast review followed by fair and prompt decisions. Once a manuscript is accepted, the outstanding editorial staff of the journal will provide high-quality support to ensure your article is the best that it can be.

We have entered an unprecedented era, in which the essence of life is increasingly understood at the molecular level, and our ability to treat disease is being improved by precision-targeted therapy. With advances in DNA-sequencing and gene-editing technologies (for example, CRISPR/Cas9), new signaling pathways and biomarkers are identified quickly, followed by the emergence of new targeted drugs and clinical treatments. *Signal Transduction and Targeted Therapy* provides a platform for basic research scientists and clinicians to share their discoveries in this field. We are committed to making the journal innovative and timely, and to providing important and valuable information to you and the community.

